# Transforming Health Care With Artificial Intelligence: Redefining Medical Documentation

**DOI:** 10.1016/j.mcpdig.2024.05.006

**Published:** 2024-05-22

**Authors:** Archana Reddy Bongurala, Dhaval Save, Ankit Virmani, Rahul Kashyap

**Affiliations:** a Pediatrics, Omni Family Health, Bakersfield, CA; b Internal Medicine, Methodist Medical Center of Illinois, Peoria, IL; c Department of Artificial Intelligence, Virufy, Los Altos, CA; d Department of Research, WellSpan Health, York, PA

The growing burden of medical documentation, particularly with the widespread adoption of electronic health records (EHRs), has contributed to physician burnout and decreased job satisfaction.^1^ The intricate structure of medical notes, combined with the time-consuming data entry process,^2^ and physicians’ often limited typing proficiency, has considerably impeded their ability to prioritize patient care.^3^ The recent rise of artificial intelligence (AI) in health care offers a promising solution to alleviate the burden of documentation and optimize clinical workflows.^4^

This article explores the transformative role of AI in medical record-keeping, highlighting its potential to reduce physician burnout and improve patient care. We explore the transition from manual record-keeping to AI-powered tools like voice-to-text transcription, data analysis, and intelligent decision aids, examining their potential to alleviate administrative tasks for doctors.

The article also examines the benefits and limitations of AI in medical charting and emphasizes the importance of balancing AI-led innovation with the preservation of health care record integrity.

### The Unintended Burden: EHRs and Clinician Burnout in the United States

In the United States, the Health Information Technology for Economic and Clinical Health Act started incentivizing hospitals, and physician practices to use EHRs in 2009.^5^ Although these systems were intended to improve care quality and efficiency, they have inadvertently contributed to clinician burnout, which is associated with higher rates of medical errors, health care costs, and clinician turnover.^6^ Electronic health record design often prioritizes billing and administrative tasks over clinical decision-making, and care delivery, leading to clinicians spending a disproportionate amount of their workday on EHR and desk work. This disrupts patient-clinician relationships, leads to inefficiencies, and increases cognitive load. The phenomenon of “note bloat,” where US physicians write notes that are 4 times longer than those in other countries and require too many steps to perform simple functions, is a substantial issue.^6^ In addition, clinicians experience a higher likelihood of burnout when they receive higher than usual automated messages in EHR inboxes.^7^

In a seminal 2017 article in the *Annals of Family Medicine*, it was reported that primary care physicians spent over 50% of their workdays on their EHRs, which averaged about 4.5 hours per day in the clinic, and 1.5 hours after hours each day at home.^8^ Nearly a quarter of this time was spent on documentation tasks within the EHR.^8^ Physicians have reported that this disproportionate time spent documenting interferes with their interactions with patients as illustrated in Figure 1, contributing to higher levels of stress and job dissatisfaction.^7^

**Figure 1:**
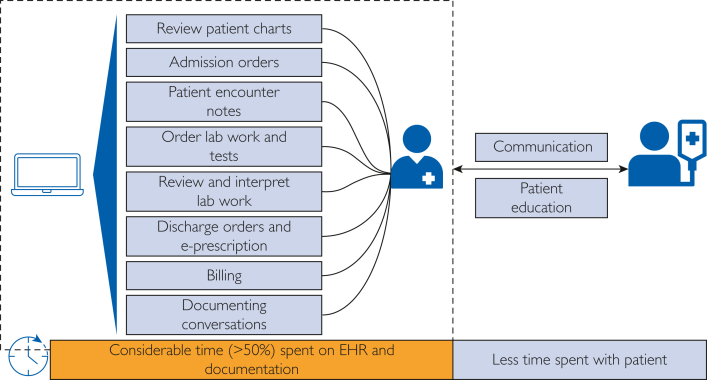
Physician’s routine tasks and responsibilities without artificial intelligence assistance. AI, artificial intelligence; EHR, electronic health record.

### Evolution of AI in Health Care

Health care is a complex field that involves numerous stakeholders and challenges, including but not limited to rising costs, limited access to care, and physician burnout.^9^ However, AI is changing health care with uses in diagnostic medicine, medication development, surgical robotics, targeted therapy, streamlining documentation, and improving physician workflow.^10^ Despite ongoing hurdles, AI for health care can considerably enhance the quality of patient care, streamline processes, and ultimately lead to better health outcomes and a more effective health care system.^11^

Early AI in health care relied on rule-based systems like MYCIN (a rule-based computer program for diagnosing infectious diseases) for tasks like bacterial identification,^12^ but limitations in adaptability hindered their progress. The evolution to pattern recognition algorithms allowed AI to learn directly from data, revolutionizing fields like medical image analysis with deep learning’s accurate disease detection^13^ and streamlining documentation with AI-powered summaries and note generation.^14^ Deep neural networks further pushed the boundaries, aiding diagnosis through analyzing complex data and subtle image abnormalities, ultimately improving accuracy and intervention timelines.^13^

### AI to the Rescue: Streamlining Documentation With AI Assistants

Strategies such as allocating work hours to tasks they find most rewarding, improving time management, and using technologies like AI-driven documentation assistants can help mitigate the burnout associated with documentation.^8^ These AI assistants can reduce a physician’s time devoted to documentation by up to 70% by transcribing patient encounters, entering data into EHRs, and processing information for orders and prescriptions, allowing physicians to focus on direct patient care.^15^

### Benefits of AI-Assisted Tools in Medical Documentation

Implementing AI and other technologies to ease the documentation burden has been explored as a way to address these issues and improve both the efficiency of clinicians and the overall health care experience. Artificial intelligence enabled medical documentation offers substantial benefits, such as automating time-consuming tasks like data entry and charting,^15^ reducing the time physicians spend on paperwork, and improving the accuracy and completeness of patient records by analyzing and interpreting large volumes of data.^15^

The landscape of AI-enabled medical documentation tools has continued to evolve, offering a wide array of innovative solutions. The implementation of natural language processing technology and predictive analytic capabilities in AI documentation tools has considerably enhanced their ability to accurately transcribe spoken words into written text, predict health concerns, and optimize treatment plans by analyzing diverse datasets.^16^

As summarized in Table, AI-powered tools are transforming health care documentation, offering a multitude of benefits:

• Reduced clinician burden: Artificial intelligence tools like voice-to-text dictation, automated notes, and summarization software markedly streamline documentation, reducing time spent on administrative tasks.^17^
• Combating burnout: Excessive documentation contributes to physician burnout. Artificial intelligence solutions alleviate this burden, potentially improving job satisfaction and physician retention.^18^
• Enhanced patient care: Reduced documentation time allows clinicians to focus on direct patient interactions, complex decision-making, and comprehensive care planning, leading to better patient experiences and potentially improved health outcomes.^19^
• Improved collaboration and quality: Artificial intelligence facilitates seamless data exchange between health care systems, enabling creation of comprehensive patient profiles, supporting collaborative care across providers, and assisting with quality initiatives.^20^
• Accurate and complete records: Artificial intelligence algorithms analyze vast amounts of data within medical records, identifying potential errors, inconsistencies, and missing details. This results in more accurate and complete patient records, enhancing the quality of care and long-term health management.^21^

**Table:** Different Types of AI-Based Tools for Charting

AI tool	Description	Strengths	Limitations
Voice-to-text dictation with AI (eg, Nuance Dragon Medical, Robin Healthcare)	Leverages natural language processing to transcribe physicians’ spoken notes directly into the EHR	Speeds up the charting process, reduces typing burden, and can include clinical decision support features	May require editing for accuracy, can struggle with accents or complex medical terminology
AI-powered chart summarization (eg, Notable and Abridge)	Analyzes patient encounters (dictations and clinical notes) to generate structured, concise summaries for easier integration into the EHR	Reduces time spent manually reviewing notes, improves efficiency in chart review, and can identify key information and potential discrepancies	May miss nuances or misinterpret context, requires physician oversight to ensure accuracy and might have privacy concerns with third-party summarization tools
Clinical documentation improvement tools (eg, 3M and Optum)	Use natural language processing and machine learning to analyze medical records for completeness and accuracy, suggesting potential diagnoses and coding improvements	Helps ensure accurate billing and reimbursement, identifies gaps in documentation for improved quality measures, and supports complex medical coding	May generate false positives or irrelevant suggestions, poses risk of overreliance, potentially leading to missed diagnoses
Virtual scribes (eg, Suki and Augmedix)	Combine AI-powered voice recognition with human scribes who review and edit transcribed notes	Offers real-time documentation support during encounters, reduces administrative burden on physicians	Costly when compared with pure AI solutions, requires physician review and finalization, and privacy concerns involving third-party scribes

Abbreviations: AI, artificial intelligence; EHR, electronic health record.

### Considerations and Areas of Improvement for AI in Charting

Artificial intelligence’s use in charting, although promising, introduces challenges. The challenges could be around data bias, security, model explainability, cost effectiveness, overalerting, and health care regulations, with more details elucidated further:

• Overreliance and deskilling: There is concern that over dependence on AI-generated notes could lead to decreased critical thinking among physicians and potentially diminish the ability to capture subtle patient cues.^22^
• Errors and misinterpretation: Artificial intelligence systems, even those with high accuracy rates, can make mistakes with medical data, especially in complex cases. Uncritical acceptance of AI suggestions carries the risk of errors in the chart.^23^
• Alert fatigue: An abundance of alerts and notifications from AI systems, including false positives, can lead to alert fatigue, causing physicians to potentially miss critical clinical information.^24^
• Bias in data and algorithms: AI models trained on biased data (eg, data sets that underrepresented minority groups) can perpetuate disparities in health care and introduce inaccuracies into medical charting.^25^
• Data privacy: Protecting patient data when using AI systems is a major concern. Huge volumes of data that are required by AI algorithms pose security related challenges in health care and could expose sensitive patient information.^26^
• Evidence of effectiveness: There is often a lack of high-quality evidence, such as randomized controlled trials, to indicate the effectiveness of AI use in real-world clinical settings.^27^
• Cost: Developing, implementing, and maintaining AI solutions can be costly, and there may be uncertainty about the return on investment for health care providers, especially in low-resource settings.^28^
• Regulatory challenges: The health care sector is highly regulated, and obtaining approval for AI applications can be a cumbersome process, which can limit the ability of these innovations to reach the market.^29^

### Solutions

To address all the aforementioned limitations and problems and to optimize AI's benefits, it is vital to approach its integration thoughtfully. Rigorous testing and validation of AI charting tools in real-world clinical scenarios are imperative to ensure accuracy, reliability, and explainability. By enabling local AI with on-device training, large language model technology can address data privacy concerns and mitigate potential biases in pretrained models, ultimately fostering trust and personalized patient education.^30^ Moreover, AI must be designed as a collaborative tool, augmenting physician judgment rather than replacing it. As elucidated in Figure 2, it is through a combination of technological advancements, regulatory foresight, interdisciplinary collaboration, and educational efforts that the health care system can use AI effectively while addressing its potential drawbacks.

**Figure 2:**
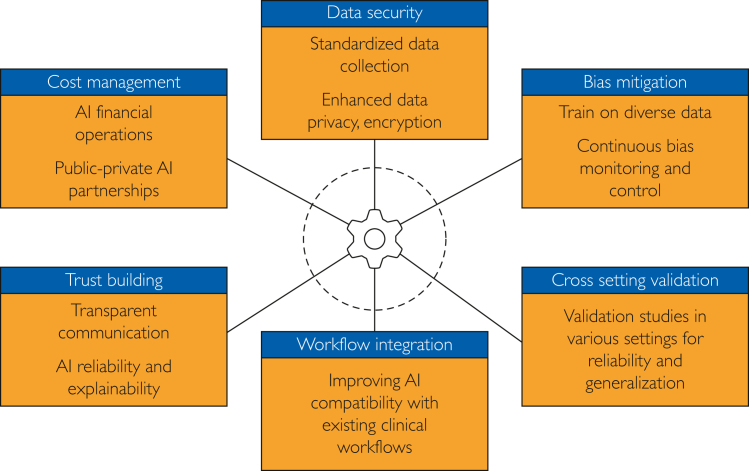
Areas of improvement for the use of artificial intelligence in charting. AI, artificial intelligence.

### Future Scope of AI in Reducing Charting Time

Artificial intelligence’s use in charting not only holds considerable promise but also presents challenges. Future AI systems need a sophisticated grasp of medical terminology to accurately interpret clinical narratives from unstructured data. This will allow AI-powered tools to generate highly accurate prepopulated chart notes, reducing manual physician input. Furthermore, AI will provide real-time clinical decision support, suggesting diagnoses, optimizing coding, and flagging inconsistencies, all of which streamline documentation and improve accuracy.^11^

Artificial intelligence’s successful integration requires addressing existing limitations and prioritizing transparency, validation, and user-centered design. Ongoing education for health care professionals about AI’s strengths and limitations is imperative for fostering trust and ensuring appropriate use.

Large language models show great promise in reducing physician charting time by automatically generating comprehensive clinical notes, summarizing patient information, and providing engaging patient education materials.^31^^,^^32^ As large language models continue to advance, their ability to understand context, capture nuances, and generate accurate and natural-sounding text will further streamline clinical documentation processes, allowing physicians to focus more on direct patient care.^31^

The future of AI in charting lies in personalization. Systems will adapt to individual physician preferences and styles, resulting in tailored chart templates and autogenerated notes. Artificial intelligence will automate repetitive administrative tasks like previous authorization requests and medication refills, freeing up physician time for valuable patient care.^10^

### Conclusion

Artificial intelligence shows a considerable promise in clinical documentation, automating burdensome tasks, streamlining workflows, improving insights, and reducing physician burnout. Thoughtfully and ethically implemented AI can transform documentation by being an intelligent assistant, liberating physicians to invest more time on patient relationship and care. While leveraging AI’s potential to improve health care, it is crucial to strike a balance between its ethical and accountable implementation, and its ability to prioritize and enhance the patient experience. The future of AI in physician documentation lies in augmenting efficiency, accuracy, and personalized patient care through automated and intelligent data processing.

## Potential Competing Interests

The authors report no competing interests.
